# Experience and Challenges in Intraocular Surgery in Patients With Kyphosis: A Cross-Sectional Survey Among Practicing Ophthalmologists

**DOI:** 10.7759/cureus.89490

**Published:** 2025-08-06

**Authors:** Sony Sinha, Prateek Nishant, Ranjeet K Sinha

**Affiliations:** 1 Ophthalmology, All India Institute of Medical Sciences, Patna, Patna, IND; 2 Ophthalmology, Employees' State Insurance Corporation (ESIC) Medical College, Bihta, Patna, IND; 3 Community Medicine, Patna Medical College, Patna, IND

**Keywords:** anterior eye segment, iatrogenic diseases, intraoperative complications, kyphosis, posterior eye segment, spinal curvatures

## Abstract

Background: The practice patterns for patient positioning, surgical techniques, and challenges faced by ophthalmologists during eye surgery on patients with kyphosis in India are yet unknown.

Methods: A cross-sectional online survey was conducted through Google Forms amongst practicing ophthalmic surgeons over two months and communicated across email lists and social media networks of state and regional ophthalmological associations of India in 2022.

Results: Fifty-two ophthalmologists responded (mean age 48.6 ± 8.5 years, 71.2% males), mostly from teaching hospitals in urban areas of eastern India. Overall, 1279 cases of kyphosis were reported to have been operated upon. The majority had 30-50° of kyphosis. Peribulbar or retrobulbar anesthesia was equally preferred with an ophthalmic operating table for positioning. Almost equal numbers of surgeons operate sitting superiorly or temporally. More than half the respondents (57.7%) reported no difficulty or complications, and 71.2% did not alter their settings while performing phacoemulsification. The majority of the remainder had problems due to the high level of the patient’s head, microscope oculars raised beyond their comfort level, and having to adjust their posture with every movement of a restless patient. Reaching the foot pedal while sitting or operating it while standing posed challenges. Surgeons commonly reported backache, neck pain, exhaustion, stiffness, tiredness, and mental stress.

Conclusions: Our study provides an Indian perspective on managing intraocular surgery in kyphotic patients with special requirements. Innovative solutions for positioning the patient and proficient surgeons can lead to the successful accomplishment of ocular surgery in such challenging patients.

## Introduction

Kyphosis is a progressive disorder of the spine that causes bowing of the back, restricted neck movement, and poor lung reserve. Such patients present obvious practical problems for ophthalmic surgeons, where supine positioning during surgery is routine [1]. In developing countries like India, patients of kyphosis with limited visual needs present with advanced cataract, glaucoma, and vitreoretinal disorders necessitating intraocular surgery. Uveitis and small pupils further compound difficulties [2]. Compromised surgical view secondary to poor positioning risks increased complications. Successful surgery in these cases requires an interdisciplinary approach with ingenuity, intuition, and resourcefulness on the part of the surgeon. Each case is unique, and surgery needs to be customised [1].

A literature search in MEDLINE and Google Scholar about the experience of ophthalmologists in India and challenges faced while performing eye surgeries on patients with kyphosis revealed isolated case reports but no studies. The present study aims to fill this knowledge gap and is the first attempt in this regard. The objective of the study is to present an Indian perspective on ophthalmic surgery in kyphotic patients.

## Materials and methods

This study comprised an online cross-sectional open survey conducted amongst practising Indian ophthalmic surgeons. The study conforms to the tenets of the Declaration of Helsinki and has been approved by the Institutional Ethics Committee. The Equator Network framework of Checklist for Reporting Results of Internet E-Surveys (CHERRIES) was adhered to.

Participants self-declared whether they fulfilled the eligibility criteria. Experience of a minimum of six months after completion of a postgraduate residency of ophthalmologists was the inclusion criterion. Those who did not perform intraocular surgery on patients with kyphosis were excluded from the study.

The investigators themselves developed the non-random questionnaire form and tested it for usability and technical functionality before opening it for collecting responses. It consisted of two parts: Part A included information regarding the competency of the surgeons and clinical settings they were employed in, and Part B asked for specific information to collect data regarding the surgeons’ experience and challenges in managing patients with kyphosis, along with space for qualitative comments and feedback as the last item in the questionnaire (Table 1). It was delivered online through Google Forms (Google LLC, Mountain View, CA) over two months and communicated across email lists and social media networks of state and regional ophthalmological associations of India in the year 2022. Reminders were sent weekly to encourage response.

**Table 1:** Questionnaire used for the study OT: operating theater; ECG: electrocardiogram

Question	Options
PART A: General Information	
Age in years	—
Gender	Male / Female / Prefer not to say
Affiliation	Teaching hospital / non-teaching hospital / Solitary private practice
Main domain of surgical practice	Cataract surgery / Cataract and refractive surgery / Corneal and refractive surgery / Vitreoretinal surgery / Glaucoma surgery / other (please mention)
Region of practice - A	North India / North-East India / East India / South India / Central India / West India
Region of practice - B	Rural area / Suburban area / Urban area
Years of active surgical practice?	—
How would you rate your proficiency in intraocular surgery?	Highly proficient / Average / Novice / Trainee
PART B: Specific Information	
How many operable cases of patients with kyphosis do you encounter in an average (non-COVID) year?	<1 / 1–5 / 6–10 / >10 / Can’t say
What is your approach to surgery in the majority of patients with kyphosis?	Operate myself in own centre / Operate in own centre but with guest surgeon / Refer to expert surgeon in another centre / Other (please mention)
How many cases of patients with kyphosis have you operated till date?	—
What is the average severity of kyphosis among your operated patients?	<30 degrees / 30–50 degrees / >50 degrees / Can’t say
How do you post your kyphotic patient in your OT list?	First / Last in the list / Middle of the list / Single patient in that day’s list
What is your preferred anesthesia?	General / Retro or peribulbar / Topical anesthesia with sedation / Topical with anxiolytic without sedation / Topical anesthesia only
What is your preferred device for surgical positioning?	OT table / Surgical chair / Patient’s chair / No preference
What modifications in your operating room or operating settings do you utilize for patient positioning?	—
What is your preferred surgeon’s position?	Superior / Temporal / Side-saddle / Face to face / Other (mention)
What is your preferred incision for anterior segment surgery?	Superior / Temporal / Inferior / Other (mention)
Do you routinely get patients with kyphosis evaluated by an orthopaedician before surgery?	Yes / No / No need, as the records are mostly available
What is the effect of such kyphosis on the surgical duration?	Slightly prolonged / Significantly prolonged (>50%) / Same as patients with no back deformity
What pre-operative difficulties have you encountered in such surgeries that you would attribute to kyphosis in these patients?	—
What complications have been encountered in such surgeries that you would attribute to kyphosis in these patients?	—
Have you ever had to abandon the surgery due to inability to position a kyphotic patient?	Yes / No / Can’t say
How do you monitor such patients during surgery?	ECG / Pulse oximeter / Blood pressure monitor / Other (please mention)
If you have performed phacoemulsification on these patients, do you recommend altering the settings on your phaco machine as compared to a routine patient?	Yes / No / Can’t say
What physical discomfort do you face during/after such a surgery?	—
Do you spend extra time explaining the possibility of complications to patients with kyphosis?	Yes / No / Can’t say
Any other comments/qualitative feedback?	—

Ophthalmologists were asked to voluntarily consent to participate by responding to the invitation to fill out the form. They were informed about the length of time of the survey, data storage, the investigator, and the purpose of the study. No personal information was collected, and no incentives were offered. All responses received within the study period were included for analysis. Missing data, if any, were reported as such.

Back-end access to the data was limited to the principal investigator and data manager, both ophthalmologists. It was automatically populated into an online spreadsheet (Google Sheets, Google LLC), which was downloaded and used for analysis. Statistical analysis was performed on SPSS version 26 (IBM Corp., Armonk, NY), wherein means and standard deviations were evaluated for continuous variables, and frequencies and proportions for categorical variables.

## Results

**Figure 1:**
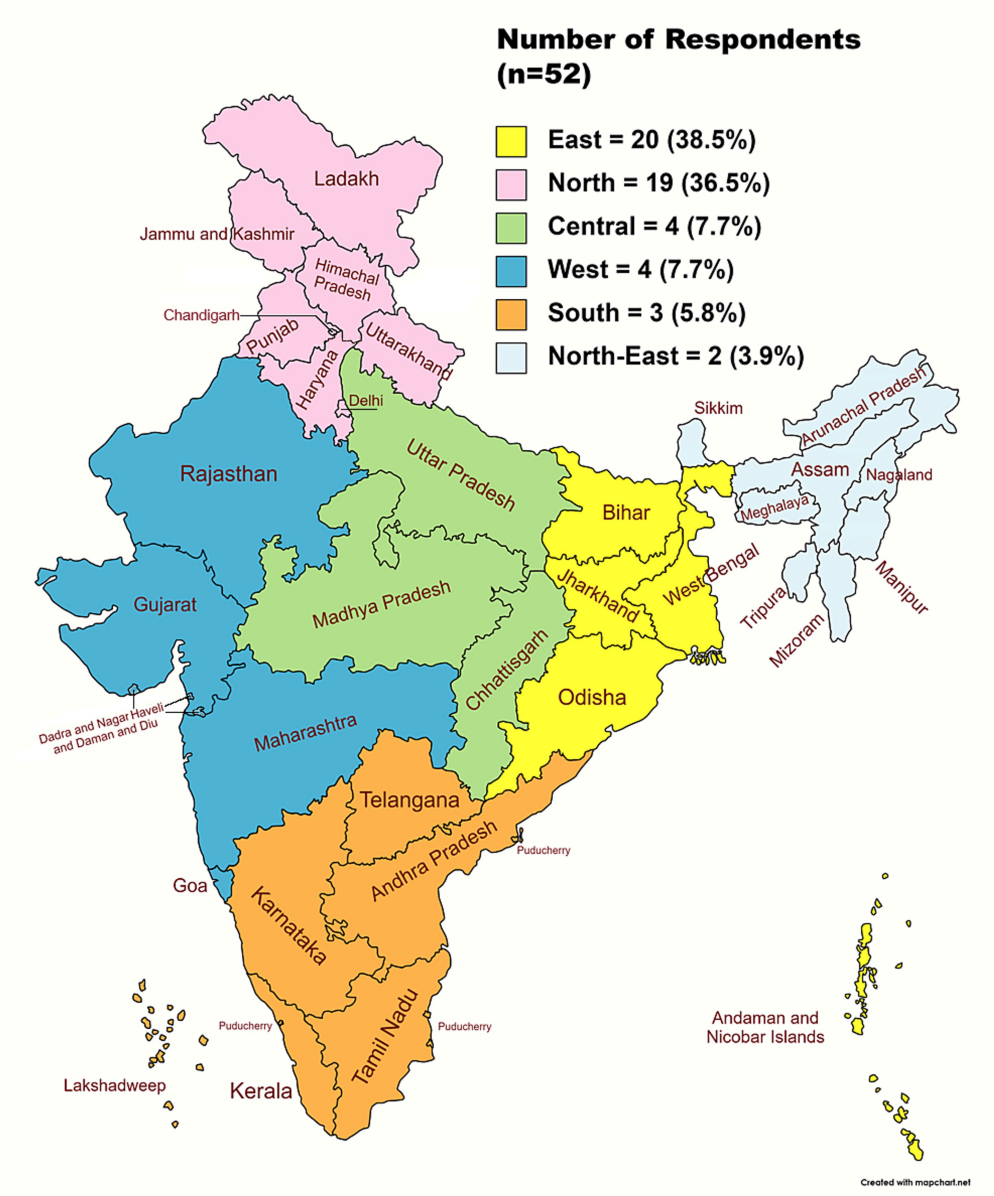
Number of respondents in our study classified as per geographical zones of India Puducherry consists of multiple non-contiguous enclaves. Created using online software www.mapchart.net and reproduced with Creative Commons CC BY-SA 4.0 DEED/Attribution-ShareAlike 4.0 International license. The representation of political boundaries does not reflect the position of the authors on issues of recognition.

Responses were received from 52 ophthalmologists (mean age 48.6±8.5 years, range 31-67 years; Figure 1).

There was a preponderance of male surgeons (71.2%), mostly from teaching hospitals (51.9%) in urban areas (84.6%). Duration of their experience as ophthalmic surgeons ranged from 2-40 years (mean 18.1±8.9 years). Most rated themselves as expert surgeons, performing predominantly cataract surgery (73.1%), including phacoemulsification and manual small incision cataract surgery. The majority of them operated on one to five such cases per year, mostly in their own center (Table 2).

**Table 2:** Demographic and competency-related particulars of participating ophthalmologists (n=52) *Multiple responses

Particulars		n	%
Gender	Males	37	71.2
	Females	15	28.8
Place of work	Teaching hospital	27	51.9
	Non-teaching hospital	12	23.1
	Private practice	13	25.0
Area of work	Urban	44	84.6
	Suburban	6	11.5
	Rural	2	3.8
Field of work*	Cataract	38	73.1
	Refractive surgery	19	36.5
	Glaucoma	6	11.5
	Vitreoretinal	5	9.6
	Cornea	3	5.8
	Oculoplasty	2	3.8
	Comprehensive	1	1.9
Level of competency	Expert	26	50.0
	Proficient	22	42.3
	Average	4	7.7

**Figure 2:**
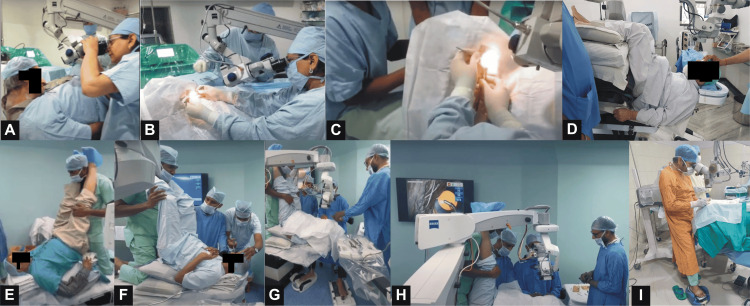
A. Trial of positioning a patient of severe upper thoracic kyphosis; B. Face-to-face phacoemulsification in the same patient; C. View over the surgeon’s shoulder to demonstrate the manner of holding the phaco probe and support by operating room staff. D. Trial of positioning a patient of severe lower thoracic kyphosis using pillows as support, achieving near-normal eye position. E. Trial of positioning a patient of moderate upper thoracic kyphosis using pillows and supported by operating room staff; F. Peribulbar anaesthesia being given to the eye; G. Odd positioning of legs leading to difficulty in controlling foot-pedal during the surgery; H. Factors enhancing the safety of surgery in this case include active fluidics, good red glow, and the scrub assistant being able to see the surgery on a monitor. I. Phacoemulsification in the standing position wherein the surgeon supports his weight on one leg. Patients' faces have been masked to conceal identity.

A cumulative experience of 1279 cases was reported (mean 24.6 cases per surgeon, range 2 to 300 cases). The majority of cases had 30-50° of kyphosis and were mostly scheduled as the last case for the day. Peribulbar/retrobulbar anesthesia was most preferred. The majority used an ophthalmic operating table for positioning, aided by pillows, towel rolls, and operating room staff. Almost equal proportions of surgeons sat superiorly or temporally (“side-saddle”). For anterior segment surgery, superior or temporal incisions were equally preferred (Figure 2, Table 3).

**Table 3:** Experience of subjects regarding intraocular surgery in patients with kyphosis (n=52) BP: blood pressure * Multiple responses

Particulars		n	%
Average cases per year	<1	14	26.9
	1-5	25	48.1
	6-10	10	19.2
	>10	3	5.8
Preferred operating setup	Own centre	48	92.3
	Refer to expert	2	3.8
	Own centre with guest surgeon	1	1.9
	Depends on severity	1	1.9
Severity of kyphosis	<30°	12	23.1
	30-50°	30	57.7
	>50°	3	5.8
	Don’t remember	7	13.5
Prior orthopaedician consultation	Do not take	38	73.1
	Take mandatorily	7	13.5
	Patient already carries it	6	11.5
	No response	1	1.9
Posting of case in OT	First case	7	13.5
	Middle case	7	13.5
	Last case	36	69.2
	Single case for the day	2	3.8
Anaesthesia	Peri/retrobulbar	36	69.2
	Topical	13	25.0
	Topical with sedation/anxiolytic	2	3.8
	No preference	1	1.9
Vitals monitoring	Pulse oximeter only	24	46.2
	Pulse oximeter and BP monitor	8	15.3
	Cardiac monitoring	8	15.3
	BP Monitor only	5	9.6
	Monitored anaesthetic care	3	5.8
	No monitoring	2	3.8
	No response	2	3.8
Patient seating	Operating table	49	94.2
	Non-surgical chair	2	3.8
	No preference	1	1.9
Position of surgeon	Superior	23	44.2
	Temporal/side-saddle	26	50.0
	Superotemporal	1	1.9
	Face-to-face	1	1.9
	No response	1	1.9
Incision location*	Superior only	24	46.2
	Temporal only	24	46.2
	Superotemporal	3	5.8
	Superior or temporal	1	1.9
	Inferior only	1	1.9

Almost three-fourths of respondents felt that their usual surgical time was prolonged. Inability of the patients to co-operate in maintaining the optimum position for surgery, restlessness, and resultant head movement were reported by five surgeons. Two surgeons felt that such actions put additional pressure to complete the surgery in a shorter time. Approaching the eye with instruments was reported as problematic by two surgeons. Four surgeons had to abandon their surgeries. However, more than half the respondents (n=30, 57.7%) reported no difficulty or complications, and a majority (n=37, 71.2%) did not alter their phacoemulsification settings (Table 4).

**Table 4:** Summary of challenges and solutions for patients with kyphosis as reported by our subjects (n=52) AETCOM: Aspects related to Attitude, Ethics and Communication; SICS: small incision cataract surgery * Multiple responses

Particulars		n	%
Pre-operative aspects			
Examination	Difficult slit-lamp examination	1	1.9
	Difficult biometry	5	9.6
AETCOM	Extra chair time	41	78.8
Intraoperative aspects			
Surgical time	Prolonged	35	67.3
	Significantly prolonged (>50%)	5	9.6
	Usual	12	23.1
Positioning	No problems	28	53.9
	Positioning of patient	10	19.2
	Holding patient by assistant	3	5.8
	Positioning microscope	7	13.5
	Positioning surgeon	7	13.5
	Standing	4	7.7
	Restless patients	5	9.6
Procedural problems in cataract surgery*	Need to be overcautious	2	3.8
	Approaching eye with instruments	2	3.8
	Rhexis	1	1.9
	Nucleus removal	1	1.9
	Small pupil	1	1.9
	Iris prolapse	1	1.9
	Vitreous upthrust	1	1.9
	Posterior capsular rent	1	1.9
	Endothelial damage/corneal edema	4	7.7
	High intraocular pressure	1	1.9
	Astigmatism	1	1.9
	Conversion to SICS	1	1.9
	Need to alter phaco settings	15	28.9
	No problems	30	57.7

Although seven surgeons (13.5%) did not respond regarding physical discomfort, the majority (n=13, 25%) had positioning problems due to the high elevation of the patient’s head, microscope oculars raised beyond their comfort level, and having to adjust their own posture with every movement of a restless patient. Reaching the foot pedal while sitting or operating it while standing posed challenges. They commonly reported neck pain (n=9, 17.3%) and backache (n=8, 15.4%), exhaustion and stiffness (n=2, 3.8% each) or tiredness and mental stress (n=1, 1.9%). Four (7.7%) surgeons did not specify details of any discomfort experienced.

## Discussion

The present study provides initial evidence regarding the practice pattern, positioning, and techniques for ophthalmic surgery adopted by Indian ophthalmologists for patients with kyphosis.

Scheduling of surgery

To the best of our knowledge, no similar study describes the preferences of surgeons to schedule challenging cases in operating lists. The majority of our respondents preferred this as the last case of the day, likely anticipating extra time needed to position the surgeon and patient, prolonged surgery, and the time needed to reorganize the operating room for subsequent cases [3].

Patient counselling

The patient is positioned as per the nature of his habitus, tolerance, and ease of access to perform surgery [4]. A comfortable patient is more likely to cooperate and tolerate the procedure [5]. Successful methods can be reused for the second eye. Sometimes, the inability to cooperate on the operating table may lead to the cancellation of a scheduled surgery [3,4,6]. Anticipating this, many respondents prefer allotting extra chair time for detailed pre-operative discussion. Special consent that the unusual positioning puts them at twice the risk of having complications, some of which may result in permanent visual impairment, is needed [3]. Detailed discussion with the surgical team and pre-operative trials of positioning are essential [7].

Patient positioning

Nearly all surgeons positioned such patients on a standard ophthalmic surgical table with pillows and towel rolls, sometimes supported by hospital staff. The most common adaptation suggested in previous studies is reclining an ophthalmic surgical chair (or a multi-part surgical table) as low as tolerable, with the face 30-80° above the horizontal [2,8]. The head may be rotated towards the surgeon in a chin-up position with the globe tilted superotemporally [2]. A recent review states that surgery is most comfortable when the patient’s eye is a little higher than the heart to achieve oculo-cerebral venous flow [9]. The use of assistance by hospital staff in holding the patient up during surgery has not been previously reported.

The Trendelenberg position is generally adopted. However, venous engorgement and elevated intraocular pressure can lead to surgical complications, while lowered pulmonary reserve due to the limitation of diaphragmatic movements and cardiovascular compromise can cause intolerance [10]. Although it may hamper access to foot pedals, this posture has been used for cataract as well as vitreoretinal surgery in patients unable to extend their neck [11,12]. Intermittent return to the horizontal position may improve patients’ tolerance [4]. Reverse Trendelenberg position achieves a chin-down posture in patients with severe kyphosis, and placement of a corneal retraction suture at 6 o'clock may help rotate the eye to the horizontal [10,13]. Our respondents did not show a preference towards either patient position.

Similar to responses received in the present study, as many as 25-30 pillows and towel rolls have been previously used to position such patients [6,13]. Tapes and straps like a parachute harness can secure the patient, with the head supported by foam pieces [4,6,11,14,15]. A wheelchair with a headrest from a surgical chair taped to it, or a standard reclining chair placed upright, has also been used [16,17]. Only two respondents reported having used a non-surgical chair in our study. Unique positioning devices like a vacuum bean bag, vacuum mattress, doughnut-shaped head and back support, modified canvas securing the patient in a vest-like manner, or other head supports have been reported [4,14,18-20]. Another innovation included an adjustable chair between two operating tables supporting the kyphotic spine, with the head on an adjacent small table with rolled towels [5]. None of our respondents used such devices for their surgeries, suggesting a lack of perceived need or poor access to them. However, a unique innovation was using operating room staff to hold patients (Figure 2).

The majority of the operating surgeons do not seek orthopaedic consultation for surgical fitness or optimum positioning; this may be related to their skills, as well as restrictions related to eye position in ophthalmic surgery.

Anesthesia and monitoring

Peribulbar or retrobulbar anesthesia was the most preferred in our study, in concordance with the UK National Survey, where only 20.7% of respondents preferred general anesthesia [21]. The latter requires the patient to be in a horizontal position to help support the endotracheal tube, which is difficult to achieve in patients with kyphosis [1]. Thus, failed face-mask ventilation and airway management difficulties can occur [4].

Sub-Tenon's anesthesia is sufficient for phacoemulsification, with only 5% of cases requiring added intravenous sedation [22]. It has been used to remove a dropped nucleus in combination with intravenous anesthesia [23]. Although pre-operative anxiolytics can be given, they are uncommonly reported in our study, probably because they alter the ability of the patient to co-operate and respond to commands [3,24]. Despite all measures, some surgeries need to be rescheduled using intravenous sedation [3].

A minority of our respondents preferred surgery under topical anesthesia. It is a known fact that this allows a cooperative patient to fixate on the microscope light for optimum visualization of the red reflex and better access [16,17]. Although monitored anesthetic care is recommended, only three respondents report using the same compulsorily, while most operate with only pulse oximeter monitoring [3].

Surgeon position

There were no specific responses about operating standing, while four respondents opined against it. A standing surgeon needs to support his weight with at least one leg, and the preference likely varies with the weight and endurance of the surgeon, type and duration of the surgery, as well as the relative height of microscope oculars [2,17,21].

Only one surgeon reported operating on a patient with severe kyphosis in the face-to-face position. This appears to be more ergonomic compared to standing, as the upper arms would be less outstretched [3,9]. The surgeon can sit very close to the patient on an adjustable small surgeon's chair [3,9,16,25,26].

Half of our respondents preferred the temporal approach with a side-saddle position wherein thighs are kept parallel to the long axis of the operating room table [7,28,29]. A temporal approach provides more room to maneuver hands and instruments as it avoids the forehead and nasal bridge, while side-saddling allows room to accommodate the legs to control foot pedals [5]. The temporal approach is preferred in eyes with small palpebral fissures and overhanging brows [14,16].

Microscope position

For extracapsular cataract extraction surgery on patients with kyphosis, surgeons have used optical loupe magnification and coaxial illumination via a headlamp [29]. For phacoemulsification with face-to-face positioning, a ceiling-mounted microscope rotated about 60° from the vertical has been used [17]. Tilting the microscope causes the surgeon's arms to be outstretched proportionately. Using the lowest level of zoom reduces the need for repeated refocusing and compensates for an obliquely placed eye. Removing cameras, assistant eyepieces, and vitreoretinal attachments may be helpful [3]. An ophthalmic technician may watch the surgeon on a monitor and reposition the microscope as required [30].

Incision

The incision is constructed according to the ability to position the eye. An equal proportion of our respondents preferred superior or temporal incisions, while only one reported having performed surgery with an inferior incision similar to a previous report of a patient with torticollis [25]. An inferior or inferotemporal approach with a clear corneal incision at 220-270° carries a theoretical risk of endophthalmitis. Still, with improved sterilization, single-use instruments, and the use of intracameral moxifloxacin, it is considerably lower.

Steps of cataract surgery

The likelihood of the speculum riding low in an upright patient can be overcome by using a screw-type Clark’s or Jaffe’s speculum or manually holding up a standard speculum. However, no such preference was reported in our study.

Capsulorrhexis was also a perceived challenge as previously reported [7]. Occasionally, incomplete rhexis required conversion to a can-opener capsulotomy. During phacoemulsification, gravity causes shallowing of the anterior chamber due to forward movement of the vitreous body and posterior capsule. It can be counteracted by using an anterior chamber maintainer, increasing the bottle height, or active fluidics [24].

The divide and conquer technique with a straight nucleus rotator has been found helpful. The unaccustomed view makes focusing and manipulation of tissues and instruments difficult. Hence, posterior capsule rents, anterior rhexis edge tears, and dropped nuclei have all been reported. Advancing the phaco-tip without damaging the posterior capsule is a challenge [7]. A backup intraocular lens (preferably a three-piece foldable lens) is a necessity. Removal of the sub-incisional cortex is also difficult. However, more than half of the surgeons in our study reported no difficulty or complications, and a majority did not alter their phacoemulsification settings. This might be because surgery in such patients is attempted mostly by highly competent surgeons.

Surgery duration and discomfort

Unusual positioning and prolonged surgery duration cause back and neck strain, more so with the advancing age of the surgeon. In our study, too, more than three-fourths had prolonged or significantly prolonged surgical time, and back and neck strain were reported by about a fifth of respondents.

Limitations of the study

This study is not without limitations. This was an online survey administered for a limited amount of time. Hence, the small sample size achieved does make it potentially underpowered. It is also noteworthy that surgeries on such patients are attempted mostly by experienced surgeons, usually working in established centers in urban areas, and not by novices in settings with limited resources. This might be another reason for the low and skewed response. The study respondents included only the surgeons who operated on patients with kyphosis; thus, there is a lack of information on the sampling frame and response rate.

Additionally, as this was a self-administered questionnaire-based study, the responses are likely subject to selection bias. Self-declaration of eligibility for the study without verification is also a limitation. Administering bias control measures in such online surveys is difficult and limits the full replicability of such studies.

The lack of validation of the questionnaire is another significant limitation. However, we could not find any similar study to obtain a validated questionnaire for this purpose. The study is based on qualitative responses and individual surgeon preferences and innovations rather than established standard practice. Hence, the inferences are not based on data and measured outcomes. Future studies might dwell on other quantitative and qualitative measures arising out of intraocular surgeries on patients of kyphosis, including baseline clinical characteristics (like nuclear density or if the other eye was pseudophakic), patient preparation time, intraoperative case data (including phacoemulsification parameters), and follow-up data (such as how many patients came back for cataract surgery in the fellow eye).

## Conclusions

Patients with kyphosis present high-risk scenarios and demand an interdisciplinary approach to accomplish ophthalmic microsurgery, which requires great precision. This is the first study that provides an Indian perspective on managing intraocular surgery in these challenging patients through a questionnaire-based survey.

In the present study, responding surgeons reported overcoming these challenges through innovative techniques in addition to surgical proficiency, leading to the successful accomplishment of intraocular surgery. Future studies involving surgeons who may have missed the current survey will help to further elucidate the practice patterns in this regard.
